# Polycystin‐1 and hydrostatic pressure are implicated in glioblastoma pathogenesis in vitro

**DOI:** 10.1111/jcmm.17212

**Published:** 2022-02-01

**Authors:** Ilianna Zoi, Antonios N. Gargalionis, Kostas A. Papavassiliou, Narjes Nasiri‐Ansari, Christina Piperi, Efthimia K. Basdra, Athanasios G. Papavassiliou

**Affiliations:** ^1^ Department of Biological Chemistry Medical School National and Kapodistrian University of Athens Athens Greece; ^2^ Department of Biopathology ‘Aeginition’ Hospital Medical School National and Kapodistrian University of Athens Athens Greece

**Keywords:** glioblastoma, hydrostatic pressure, mechanobiology, polycystin‐1

## Abstract

The mechanobiological aspects of glioblastoma (GBM) pathogenesis are largely unknown. Polycystin‐1 (PC1) is a key mechanosensitive protein which perceives extracellular mechanical cues and transforms them into intracellular biochemical signals that elicit a change in cell behaviour. The aim of the present study was to investigate if and how PC1 participates in GBM pathogenesis under a mechanically induced microenvironment. Therefore, we subjected T98G GBM cells to continuous hydrostatic pressure (HP) and/or PC1 blockade and evaluated their effect on cell behaviour, the activity of signalling pathways and the expression of mechano‐induced transcriptional regulators and markers associated with properties of cancer cells. According to our data, PC1 and HP affect GBM cell proliferation, clonogenicity and migration; the diameter of GBM spheroids; the phosphorylation of mechanistic target of rapamycin (mTOR), extracellular signal‐regulated kinase (ERK) and focal adhesion kinase (FAK); the protein expression of transcription cofactors YES‐associated protein (YAP) and transcriptional coactivator with PDZ‐binding motif (TAZ); and the mRNA expression of markers related to anti‐apoptosis, apoptosis, angiogenesis, epithelial to mesenchymal transition (EMT) and proliferation. Together, our in vitro results suggest that PC1 plays an important role in GBM mechanobiology.

## 1 INTRODUCTION

Mechanotransduction is a key biological process whereby cells convert extracellular mechanical cues into intracellular biochemical signals, resulting in the regulation of cell phenotype and behaviour. In the context of cancer, mechanical forces are continuously applied within the tumour microenvironment. Mechanical changes in the microenvironment of brain tumours include increased pressure due to oedema, cellular compression, stiffening of the extracellular matrix (ECM), increased cellular contractility and pressure applied to the cell membrane, all of which can favour gliomagenesis by triggering the activation of mechano‐induced oncogenic signalling pathways.^1^, ^2^, ^3^, ^4^, ^5^, ^6^, ^7^

In gliomas, it appears that the stiffness of the ECM progressively increases from low‐grade to high‐grade tumours. Glioblastoma (GBM), which is the most aggressive glioma, exhibits the greatest stiffness despite its heterogeneous nature. Importantly, this increased stiffness is associated with a worse prognosis.^4^ Progressive transformation from low‐grade glioma to GBM is also accompanied by enhanced mechanotransduction, as evidenced by the phosphorylation of mechanically activated proteins, such as focal adhesion kinase (FAK) and myosin regulatory light chain 2 (MLC2).^4^ Gliomas with *isocitrate dehydrogenase* (*IDH*) mutations, which have a better prognosis, are characterized by less stiffness than tumours with wild‐type *IDH*, which are more rigid and present with a worse prognosis. In the latter, increased expression of structural components that increase the hardness of ECM is detected, including hyaluronic acid and tenascin‐C protein (TNC).^4^ The median pressure, generated by the pressure difference that exists between normal tissue and tumour, has also been shown to increase the shear forces of the extracellular fluid, promoting the local invasion of glioma cells.^3^, ^5^

Polycystins have emerged as major mechanosensitive molecules in epithelial cells. They represent a family of proteins consisting of 8 protein molecules. The two representative members of the family are polycystin‐1 (PC1) and polycystin‐2 (PC2), which are normally detected in most tissues of the human body. The proteins are encoded by the *PKD1* (*Polycystic Kidney Disease 1*) and *PKD2* (*Polycystic Kidney Disease 2*) genes, located on chromosomes 16p13.3 and 4q21‐23, respectively.^8^ Mutations in these genes cause autosomal dominant polycystic kidney disease (ADPKD), the most common inherited kidney disease.^9^

PC1 is a transmembrane protein with a large and flexible amino (N)‐terminus, and a carboxy (C)‐terminus that produces transcriptionally active fragments. It functions as a mechanosensory molecule that detects extracellular mechanical stimuli. As an atypical G protein‐coupled receptor it can modulate cellular responses that include proliferation, differentiation and apoptosis.^10^ PC1 is located in multiple focal adhesion structures, the primary cellular structures that mediate cell communication with ECM.^11^ PC2 is a non‐selective cation channel permeable to calcium ions, which belongs to the family of transient receptor potential (TRP) channels.^12^ In terms of their expression in the human brain, high levels of PC1 are found in the cerebral cortex.^13^ Specifically, PC1 presents high expression in astrocytes relative to precursor nerve cells (20‐fold higher).^14^ PC2 is diffusely expressed in several neural tissues, particularly in the neural tube and nerve ganglia, while in the sixteenth week of organogenesis its expression is more pronounced in the anterior roots of the spinal cord.^15^ As far as the role of polycystins in cancer is concerned, they have already been identified as mechanosensitive proteins involved in the biology of various types of cancer, such as colorectal cancer (CRC), renal cell carcinoma, prostate cancer, breast cancer, lung cancer and GBM.^16^, ^17^, ^18^, ^19^, ^20^, ^21^ In GOS‐3 GBM cells, PC1 has been reported to regulate cancer cell behaviour and to interact with mechanistic target of rapamycin (mTOR) and Janus kinase (JAK) signalling pathways.^19^

All the above data led us to the hypothesis that polycystins play a significant role in the development and progression of gliomas. Therefore, the aim of the present study was to probe the potential role of mechanosensitive PC1 in GBM pathogenesis in relation to mechanical stimulation.

## 2 MATERIALS AND METHODS

### 2.1 Cell cultures

GOS‐3 and T98G cancer cells were kindly provided by Dr. Robert W. Lea, Department of Biological Sciences, University of Central Lancashire. The cell lines were cultured in RPMI 1640 medium GlutaMAX supplemented with 10% foetal bovine serum (FBS) and 1% penicillin/streptomycin (10,000 U/ml penicillin‐10,000 µg/ml streptomycin). Cell cultures were maintained at 37°C in a humidified atmosphere containing 5% CO_2_‐95% air. Cells were treated with the inhibitory IgPC1 antibody (dilution 1:50) for 24, 48 and 72 h in medium. The IgPC1 antibody was a generous gift from Dr. Oxana Ibraghimov‐Beskrovnaya and Dr. Herve Husson (Genzyme).

### 2.2 Human tissue

A sample of adult normal brain (cerebellum) tissue (both as preserved specimen and in paraffin blocks) was used in the present study and was obtained from the archives of the First Department of Pathology, “Laikon” General Hospital, Medical School, National and Kapodistrian University of Athens.

### 2.3 Hydrostatic pressure (HP) apparatus

T98G cells were cultured in cell plates or dishes depending on the assay applied. When cells reached the appropriate confluency, they were placed into the chamber of the “Continuous Flow Constant Pressure” hydrostatic pressure system ( “Continuous flow constant pressure for cell culture” apparatus, designed and developed by Inspiration Technology Innovation, ITI, Athens, Greece, http://iti.com.gr/)^22^ and 100 g/cm^2^ of continuous hydrostatic pressure (HP) was delivered to the cell monolayer. Following application of HP, cells were harvested immediately.

### 2.4 *PKD1* knockdown

Prior to transfection, cells were starved for 6 h in order to achieve proper cell cycle synchronization. T98G cancer cells were transfected overnight with Dharmacon's chemically synthesized siRNA SMARTpools [human PC‐1, L‐007666‐00‐0005, ON‐TARGETplus Human PKD1 (5310) siRNA–SMARTpool, 5 nmol] and non‐targeting siRNA for control cells (D‐001210‐01‐05, siGENOME Non‐Targeting siRNA #1, 5 nmol), in dilution 1:20 in 1× siRNA buffer, using DharmaFECT 2 Transfection Reagent, 0.2 ml (Dharmacon) in dilution 1:50 in DMEM (Gibco, Thermo Fisher Scientific).^18^, ^19^ After 16 h, the medium was changed and the cells were treated with IgPC1 and/or HP and cultured for 24 and 48 h.

### 2.5 Antibodies

The following primary antibodies were used for Western blot analysis: polycystin‐1 (sc‐130554, Santa Cruz Biotechnology), polycystin‐2 (sc‐10376, Santa Cruz Biotechnology), ERK1/2 (sc‐514302, Santa Cruz Biotechnology), phospho‐ERK1/2 (Abcam ab32538), mTOR (701483, Thermo Fisher Scientific), phospho‐mTOR (5536 CST), FAK (sc‐271126, Santa Cruz Biotechnology), phospho‐FAK (8556 CST), YAP (sc‐101199, Santa Cruz Biotechnology), TAZ (sc‐518026, Santa Cruz Biotechnology) and actin (MAB1501, Millipore). The following secondary antibodies were used: goat anti‐mouse IgG HRP‐conjugate (AP124P, Millipore), goat anti‐rabbit IgG HRP‐conjugate (AP132P, Millipore) and donkey anti‐goat IgG HRP‐conjugate (A00178, GenScript).

### 2.6 Semi‐quantitative PCR and quantitative real‐time PCR

Total RNA was extracted from T98G cells using RNeasy Mini Kit (Qiagen, Cat No: 74134). The concentration and quality of extracted mRNA were evaluated by a NanoDrop™ instrument (Thermo Scientific). All the extracted RNA (1000 ng) was reverse transcribed into cDNA using PrimeScript RT reagent kit (Takara‐RR037A).

For semi‐quantitative PCR, the produced cDNA was amplified with specific primer pairs for PC1‐encoding *PKD1* (annealing 58°C, forward CGCCGCTTCACTAGCTTCGAC; reverse ACGCTCCAGAGGGAGTCCAC) and PC2‐encoding *PKD2* (annealing 53°C, forward GCGAGGTCTCTGGGGAAC; reverse TACACATGGAGCTCATCATGC) genes (35 cycles) as well as with *ACTB* gene primer pairs (28 cycles) using KAPA2G Fast Multiplex PCR Kit (KK5801, Kapa Biosystems). PCR‐amplified fragments were analysed after their separation in agarose gels using image analysis software (Image J) and normalized to actin gene levels.

RT‐PCR product was amplified using the Quanti Nova SYBR Green PCR Kit (Qiagen, Cat No: 208054) in a total reaction volume of 20 μl and on a CFX96 (Bio‐RAD). The primer efficiencies were calculated from a standard curve of serially diluted cDNA. A melting curve analysis was performed to confirm the specificity of quantitative polymerase chain reaction (qPCR) products. Fold changes were calculated using the 2^–∆∆Ct^ method, and all values were normalized against GAPDH and relative to the untreated control (mock). Differentially expressed genes were identified through fold change filtering where a minimum of twofold change was considered significant. All reactions were performed in triplicates and repeated at least three times. The sequences of primers used for the amplification of *CDH2* (N‐cadherin‐encoding) were as follows: forward primer: 5′‐CTCCTATGAGTGGAACAGGAACG‐3′ and reverse primer: 5′‐ TTGGATCAATGTCATAATCAAGTGCTGTA‐3′; for the amplification of *SNAI1* forward primer: 5′‐GAGGCGGTGGCAGACTAG‐3′ and reverse primer: 5′‐ GACACATCGGTCAGACCAG‐3′; for the amplification of *SNAI2* forward primer: 5′‐CATGCCTGTCATACCACAAC‐3′ and reverse primer: 5′‐GGTGTCAGATGGAGGAGGG‐3′; for the amplification of *MKI67* (Ki‐67‐encoding) forward primer: 5′‐CTTTGGGTGCGACTTGACG‐3′ and reverse primer: 5′‐GTCGACCCCGCTCCTTTT‐3′, for the amplification of *BCL2* forward primer: 5′‐GCTGAAGATTGATGGGATCG‐3′ and reverse primer: 5′‐TACAGCATGATCCTCTGTCAAG‐3′, for the amplification of *BAX* forward primer: 5′‐CCGCCGTGGACACAGAC‐3′ and reverse primer: 5′‐ CAGAAAACATGTCAGCTGCCA‐3′, for the amplification of *VEGFA* forward primer: 5′‐AGGGCAGAATCATCACGAAG‐3′ and reverse primer: 5′‐ CACACAGGATGGCTTGAAGA‐3′ and for the amplification of *PKD1* forward primer 5′‐CAAGACACCCACATGGAAACG‐3′ and reverse primer 5′‐CGCCAGCGTCTCTGTCTTCT‐3′.

### 2.7 Western blot analysis

Proteins were resolved by electrophoresis in SDS‐polyacrylamide gels with varying densities (6% for PC1; 8% for mTOR and p‐mTOR; 10% for PC2, FAK and p‐FAK; 12% for ERK1/2, p‐ERK1/2, YAP, TAZ and actin) and transferred to a nitrocellulose membrane (Macherey‐Nagel). Membranes were blocked for 1 h at room temperature in Tris‐buffered saline with Tween‐20 (TBS‐T) with 5% nonfat milk. Then, membranes were incubated overnight at 4°C with the primary antibodies (dilutions were 1:250 for antibodies against PC1, PC2, mTOR; 1:500 for ERK1/2, FAK, YAP, TAZ and p‐ERK1/2; 1:1000 for p‐mTOR, p‐FAK, actin in TBS‐T containing 5% BSA). After incubation with HRP‐conjugated secondary antibodies, the detection of the immunoreactive bands was performed with the Clarity Western ECL Substrate (Bio‐Rad). Relative protein amounts were evaluated by a densitometry analysis using ImageJ software and normalized to the corresponding actin levels.

### 2.8 Cell proliferation assay

Cells were seeded in a 96‐well plate at a density of 10^3^–10^5^ cells/well in 100 μl of culture medium with IgPC1 (1:50 dilution) or non‐immune rabbit serum. Cells were cultured in a CO_2_ incubator at 37°C under HP for 24 and 48 h. 10 μl of the prepared XTT Mixture (XTT Cell Proliferation Assay Kit, 10010200; Cayman Chemical) was added to each well and mixed gently. The cells were incubated for 2 h at 37°C in a CO_2_ incubator. The absorbance of each sample was measured using a microplate reader at a wavelength of 450 nm. Cells were synchronized with serum starvation for 6 h.

### 2.9 Cell migration assay

T98G cells were cultured in 12‐well cell plates until confluent and synchronized by serum starvation for 6 h. The cellular layer was etched with a 200 μl sterile pipette tip. Cells were treated with IgPC1 or non‐immune rabbit serum and/or HP. Each location was photographed in a computer‐connected microscope at 0 h and after a 24‐ and 48‐h incubation. Images were analysed using TScratch software. The results were expressed as percentages of the cell‐coated region (wound recovery %).

### 2.10 Clonogenic assay

Cancer cells were seeded in 6‐well plates, at an appropriate seeding density (~10^3^ cells/well). Cells were allowed to attach to the wells and then were treated with IgPC1 and/or HP. Plates were placed in a CO_2_ incubator at 37°C for 10–15 days, until control cells formed sufficiently large colonies. Cells were then fixed with a solution containing 1 acetic acid: 7 methanol and stained with 0.5% crystal violet in methanol for 15 min. Plates were carefully immersed in a tank with tap water and left to dry. Next, they were scanned, and the relative capacity to produce colonies was evaluated by a densitometry analysis using ImageJ software.

### 2.11 Hanging drop cell culture for generation of spheroids

Using a 20‐μl pipettor, 10 μl of 2.5 × 10^6^ T98G cells/ml was deposited onto the bottom of the lid of a 10‐cm tissue culture dish. The lid was placed back to the dish filled with 15 ml sterile PBS, and cells were incubated at 37°C, 5% CO_2_–95% humidity. The drops were incubated for 2–3 days until aggregates formed. Following, spheroids were transferred to low‐adherence plates and treated with IgPC1 and/or HP for 4 days. Each spheroid was photographed in a computer‐connected microscope at days 1, 2, 3 and 4. Spheroid diameter (μm) was measured using ZEN 2 software.

### 2.12 Statistical and image analysis

All experiments were performed at least three times. Data are presented as mean ± SD and were analysed by one‐way ANOVA. GraphPad Prism 6 software was employed for these statistical analyses. All statistical tests were two‐sided. *p* < 0.05 was considered statistically significant.

## 3 RESULTS

### 3.1 PC1 and PC2 expression in GBM cell lines and normal brain tissue

**FIGURE 1:**
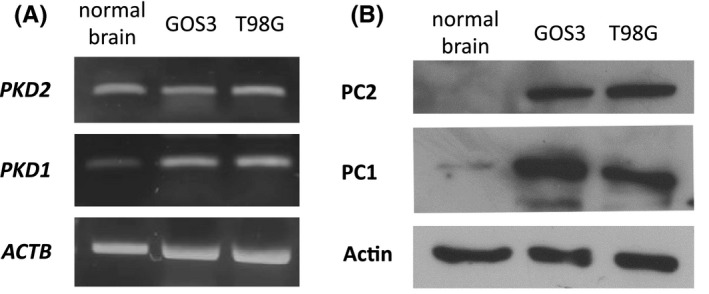
PC1 and PC2 expression in GBM cell lines and normal brain (cerebellum) archival tissue. (A) PCR electrophoresis showing RNA expression of *PKD1* and *PKD2* in normal brain tissue, GOS‐3 and T98G cell lines. (B) Western blot showing protein expression of PC1 and PC2 in normal brain tissue, GOS‐3 and T98G cell lines

Initially, the RNA and protein expression of both polycystins, PC1 and PC2, were identified in GBM cell lines GOS‐3 and T98G, as well as in normal brain tissue (Figure 1A,B). While PC1 and PC2 present marginal protein expression in normal brain, they are firmly expressed in GBM cells (Figure 1B), indicating a potential implication of these two proteins in GBM pathogenesis. Of note, the presence of polycystins RNA in combination with the absence of protein expression in normal brain implies strong post‐transcriptional regulation.

### 3.2 Impact of HP and/or PC1 blockade on proliferation, clone formation, and migration of GBM cells

**FIGURE 2:**
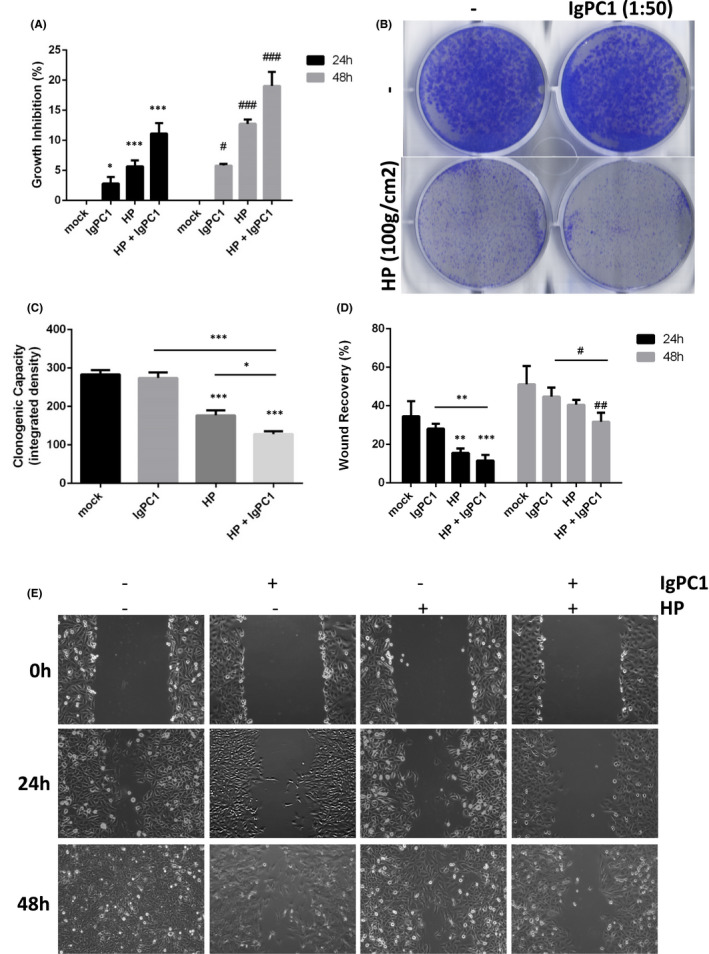
Impact of hydrostatic pressure (HP) and/or PC1 blockade on proliferation, clone formation and migration of GBM cells. (A, B, C) Quantification charts and T98G cells aggregates showing growth inhibition and clonogenic capacity in untreated T98G cells, cells treated with IgPC1, cells under hydrostatic pressure (HP) and cells under both treatments at 24 and 48 h. (D, E) Quantification chart and wound healing microscopy images showing wound recovery in untreated T98G cells, cells treated with IgPC1, cells under HP and cells under both treatments at 24 and 48 h (*p < 0.05, **p < 0.01, ***p < 0.001)

In order to investigate the mechanobiological aspect of GBM pathogenesis in vitro, we chose to subject GBM cells to mechanical stimulation by applying HP to T98G cell cultures. Additionally, since PC1 mechanical sensitivity is mediated through its N‐terminal extracellular end, GBM cells were also incubated with the IgPC1 antibody that binds to and functionally blocks the PC1 N‐terminal end, resulting in loss of PC1 mechanosensitivity. A proliferation and clonogenic assay were performed to evaluate the potential growth inhibition and clonogenic capacity of GBM cells, respectively, under HP and/or PC1 blockade (Figure 2A–C). We observed that PC1 blockade or HP suppressed the clonogenic capacity and inhibited the growth of GBM cells, with the latter occurring at 24 and 48 h. Notably, there appears to be a synergistic effect of HP with PC1 blockade as simultaneous application of both further decreased clonogenicity and enhanced growth inhibition in GBM cells at both time points (Figure 2A–C). A wound healing assay was also performed to evaluate the effect of HP and/or PC1 blockade on GBM cell migration at 24 and 48 h (Figure 2D,E). GBM cell migration decreased either under HP or PC1 blockade alone at both time points, with the negative effect of HP at 24 h being statistically significant. Similarly to the previous assays, we observed a synergistic effect of HP with PC1 blockade since combined treatment further decreased cell migration in GBM cells at 24 and 48 h (Figure 2D,E). Taken together, these data indicate that HP and PC1 affect GBM cell behaviour, with HP impeding GBM proliferation, migration and clonogenicity, while PC1 promotes these oncogenic processes.

### 3.3 Impact of HP and/or PC1 blockade on GBM spheroid diameter

**FIGURE 3:**
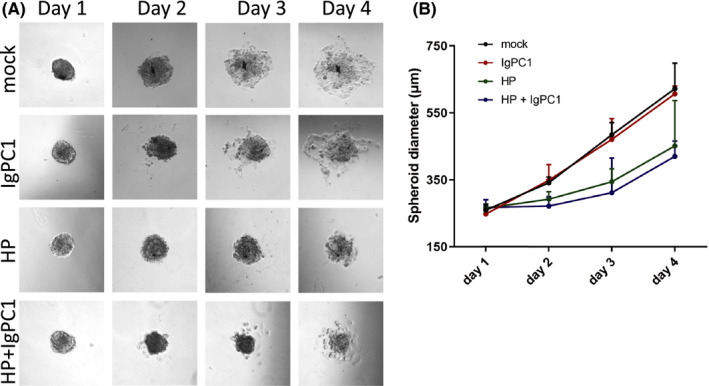
Impact of hydrostatic pressure (HP) and/or PC1 blockade on GBM spheroid diameter. Microscopy (A) and diagram (B) showing spheroid diameter at days 1–4 in untreated T98G cells, cells treated with IgPC1, cells under hydrostatic pressure (HP) and cells under both treatments

Next, we generated T98G spheroid cultures in order to overcome the limitations of monolayer cell culture and create an optimized physicochemical environment in vitro. 3‐dimensional (3‐D) cultures were subjected to HP and/or PC1 blockade, and the diameter of T98G spheroids was assessed under all treatment conditions. Untreated GBM cells had a similar diameter to GBM cells that were treated with IgPC1. Application of HP resulted in a notable decrease of GBM spheroid diameter, while GBM spheroids under both HP and PC1 blockade exhibited a further decrease of their diameter (Figure 3). These data suggest that in a 3‐D GBM culture model, when PC1 is blocked GBM cells are not able to efficiently interact with each other and with the ECM, resulting in a loose spheroid formation. On the other hand, HP promotes spheroid formation, triggering GBM cells to become more compact in a 3‐D culture. Surprisingly, treatment of GBM spheroids with both IgPC1 and HP enhanced this effect.

### 3.4 Impact of HP and/or PC1 blockade on mTOR, FAK and ERK phosphorylation in GBM cells

Since PC1 and HP can regulate GBM cell behaviour and spheroid diameter, we next sought to gain insight into the molecular mechanisms of these effects and identify potential downstream signalling pathways that are activated/regulated/modulated in response to HP and/or PC1 mechano‐stimulation. We focused on the phosphorylation status of mTOR because of its association with PC1 in the context of ADPKD and cancer, including GBM, and FAK and ERK as these proteins have been previously associated with PC1, mechanobiology and GBM.^11^, ^17^, ^18^, ^19^, ^23^, ^24^, ^25^, ^26^, ^27^, ^28^, ^29^, ^30^ T98G cells were treated with HP and/or IgPC1 for 24, 48 and 72 h. Following harvesting of GBM cells, the levels of phosphorylated and total forms of mTOR, FAK and ERK were measured.

**FIGURE 4:**
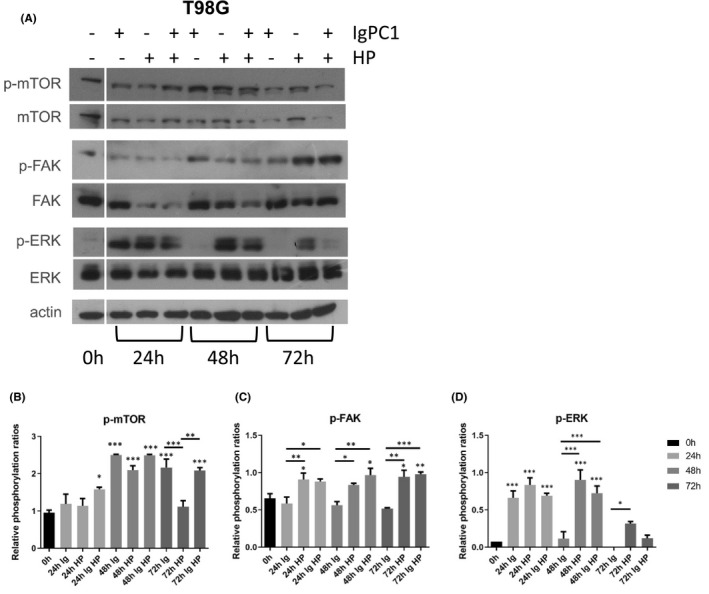
Impact of hydrostatic pressure (HP) and/or PC1 blockade on mTOR, FAK and ERK phosphorylation in GBM cells. (A) Western blots showing expression of phospho‐mTOR, mTOR, phospho‐FAK, FAK, phospho‐ERK, ERK in untreated T98G cells, cells treated with IgPC1, cells under hydrostatic pressure (HP) and cells under both treatments at 0, 24, 48 and 72 h. (B) Quantification chart showing relative phosphorylation ratios of mTOR. (C) Quantification chart showing relative phosphorylation ratios of FAK. (D) Quantification chart showing relative phosphorylation ratios of ERK (*p < 0.05, **p < 0.01, ***p < 0.001). Notes: (i) the actin bands in Figures 4A and 5 are identical to each other because the samples ran in both cases are identical and have the same quantity of proteins, thus, the same actin bands were used in both figures for data normalization; (ii) the space between the first and the other lanes, indicative of slicing, exists because, originally, we also evaluated protein expression under IgPC1 and/or HP for 12 h (a time point not providing valuable information for the interpretation of our data)

mTOR was phosphorylated in response to either HP, PC1 blockade or combined treatment at 48 h. mTOR phosphorylation was also prominent when T98G cells were treated for 24 h with IgPC1 and HP, as well as when T98G cells were treated for 72 h with IgPC1 alone and the combination of IgPC1 with HP (Figure 4A,B). FAK phosphorylation increased at a significant level at 24 h under HP alone, at 48 h under combined treatment and at 72 h under both HP alone and combined treatment (Figure 4A,C). ERK phosphorylation was almost absent at 0 h; however, it notably increased at 24 and 48 h following application of HP alone or both HP and IgPC1. Individually, PC1 blockade triggered ERK phosphorylation at 24 h, but not at later time points during the experiment (Figure 4A,D). The positive effect of the combined treatment on the phosphorylation of mTOR at 72 h, of FAK at all time points, and of ERK at 48 h was significantly greater than the phosphorylation of mTOR at 72 h when only HP was applied, of FAK at all time points, and of ERK at 48 h when only IgPC1 was applied, respectively. All these results suggest that PC1 and HP may regulate the activation of mTOR, FAK and ERK in GBM cells in a time‐dependent manner.

### 3.5 Impact of HP and/or PC1 blockade on the expression of mechano‐induced transcription cofactors in GBM cells

**FIGURE 5:**
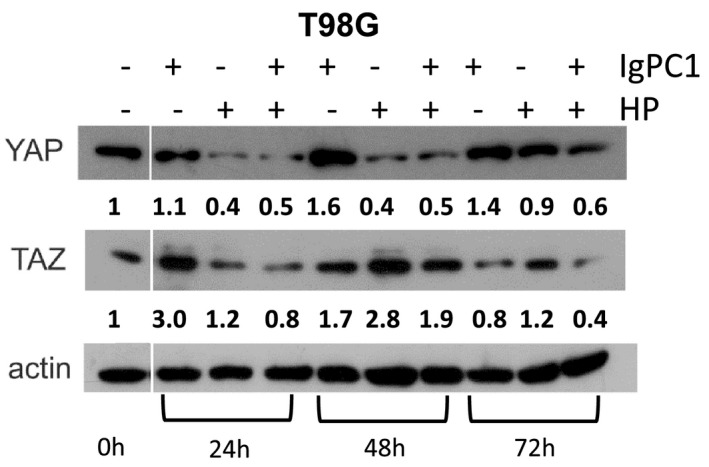
Impact of hydrostatic pressure (HP) and/or PC1 blockade on the expression of mechano‐induced transcription cofactors YAP and TAZ in GBM cells. Western blots showing expression of transcription cofactors YAP and TAZ in untreated T98G cells, cells treated with IgPC1, cells under hydrostatic pressure (HP) and cells under both treatments at 0, 24, 48 and 72 h. The numbers below the YAP and TAZ blots indicate relative YAP and TAZ levels after normalization. A representative experiment is shown. Notes: (i) the actin bands in Figures 4A and 5 are identical to each other because the samples ran in both cases are identical and have the same quantity of proteins; thus, the same actin bands were used in both figures for data normalization; (ii) the space between the first and the other lanes, indicative of slicing, exists because, originally, we also evaluated protein expression under IgPC1 and/or HP for 12 h (a time point not providing valuable information for the interpretation of our data)

Based on our previous results regarding the modulation of intracellular signalling pathways by PC1 and HP in GBM cells, we wondered whether PC1 blockade and/or HP may also affect the levels of proteins involved in the regulation of gene transcription within the GBM cell nucleus. Specifically, we decided to focus on two well‐known mechano‐induced transcription cofactors, namely, YAP and TAZ because both have been identified as mediators of PC1‐dependent mechanotransduction^31^, ^32^, ^33^, ^34^, ^35^ and are implicated in GBM pathogenesis.^36^ Therefore, we investigated the protein levels of YAP and TAZ in T98G cells following PC1 blockade and/or application of HP for 0, 24, 48 and 72 h (Figure 5). When T98G cells were treated only with IgPC1, the protein expression of both YAP and TAZ increased at all time points except for the expression of TAZ at 72 h which showed a slight decrease compared to control cells. While the protein levels of YAP decreased at all time points under HP alone, the protein levels of TAZ displayed an increase at all time points in comparison with control cells. The combined application of both IgPC1 and HP led to a downregulation of YAP and TAZ expression at all time points, except for the expression of TAZ at 48 h which was upregulated. According to these findings, PC1 and HP can regulate the protein expression of transcription cofactors YAP and TAZ in GBM cells and this regulation is time‐dependent.

### 3.6 Impact of HP and/or PC1 blockade on the expression of apoptotic, angiogenic, EMT and proliferation markers

**FIGURE 6:**
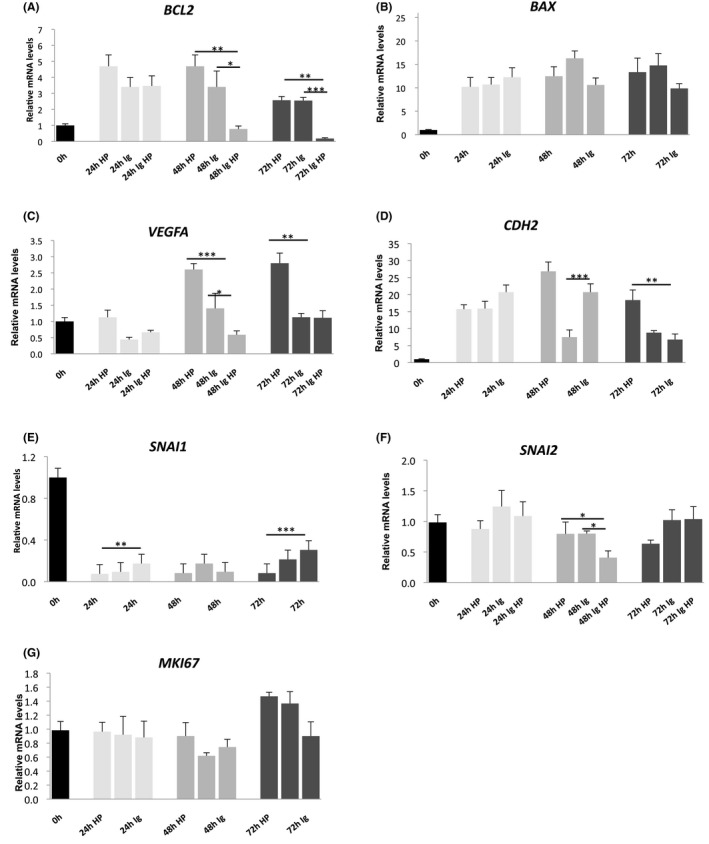
Impact of hydrostatic pressure (HP) and/or PC1 blockade on the expression of apoptotic, angiogenic, EMT and proliferation markers in GBM cells. Quantification charts of qPCR showing mRNA expression of *BCL2* (A), *BAX* (B), *VEGFA* (C), *CDH2* (*N‐cadherin*) (D), *SNAI1* (E), *SNAI2* (F) and *MKI67* (G) in untreated T98G cells, cells treated with IgPC1, cells under hydrostatic pressure (HP) and cells under both treatments at 0, 24, 48 and 72 h (*p < 0.05, **p < 0.01, ***p < 0.001)

The observed effect of PC1 and HP on cell behaviour, the activation of signalling pathways and the protein expression of transcriptional regulators in GBM cells prompted us to explore if certain tumour‐associated biomarkers are also affected when PC1 is blocked and/or HP is applied. Thus, we employed quantitative real‐time PCR analysis to evaluate the expression of the anti‐apoptotic *BCL2*, pro‐apoptotic *BAX*, pro‐angiogenic *VEGFA*, EMT‐promoting *CDH*, *SNAI1* and *SNAI2*, and proliferation‐associated *MKI67* gene at 0, 24, 48 and 72 h of treatment with IgPC1 and/or HP (Figure 6). The *BCL2* mRNA levels were elevated after IgPC1 or HP treatment at all time points. Whereas combined treatment decreased the mRNA levels of *BCL2* at 48 and 72 h, at 24 h, it resulted in increased mRNA levels of *BCL2* (Figure 6A). *BAX* and *CDH* mRNA expression increased in response to IgPC1 and/or HP treatment at all time points compared to control (Figure 6B,D, respectively). The mRNA expression of *VEGFA* was substantially upregulated under HP at 48 and 72 h, while treatment with IgPC1 caused *VEGFA* mRNA expression to increase at 48 h and decrease at 24 h. Combined treatment for 24 and 48 h caused *VEGFA* mRNA levels to decrease (Figure 6C). *SNAI1* mRNA expression was significantly decreased under all treatments at all time points (Figure 6E). This expression pattern was not observed for *SNAI2* which decreased under all treatments only at 48 h. When GBM cells were exposed to IgPC1 alone for 24 h, their *SNAI2* mRNA was increased. Application of HP alone for 72 h led to decreased *SNAI2* mRNA levels (Figure 6F). Finally, the mRNA expression of *MKI67* was downregulated following treatment with IgPC1 and/or HP for 48 h, whereas after treatment with HP or IgPC1 for 72 h it was upregulated. These results imply that PC1 and HP regulate the expression of proteins that are involved in the acquisition of various cancer cell‐related traits, including apoptosis, anti‐apoptosis, angiogenesis, EMT and proliferation.

## 4 DISCUSSION

Gliomas represent the most common primary tumour of the central nervous system and are associated with a particularly high mortality and morbidity.^37^ Several factors have been shown to play a role in GBM pathogenesis, with cancer tissue mechanics emerging as a key contributor to its development and progression. Abnormal ECM stiffness and aberrant mechanotransduction can promote glioma growth. In addition, HP has been shown to increase GBM invasiveness.^6^ The response of glioma cells to increased mechanical pressure involves mechanosensitive protein molecules such as Piezo1, talin‐1, caveola‐forming proteins, tenascin‐c and Rac1.^1^, ^2^, ^4^, ^6^, ^7^, ^38^, ^39^ Furthermore, characterization of the nano‐mechanical properties of GBM provides a useful tool for distinguishing normal from malignant brain tissue.^40^

Polycystins are mechanosensitive proteins that have been associated with the biology of certain solid tumours. Their effect on cancer appears to be context‐dependent. In CRC, PC1 overexpression promotes EMT and PC2 overexpression induces mTOR pathway activation. Increased expression of both PC1 and PC2 has also been associated with aggressive phenotypes of CRC cells, while PC1 has emerged as an independent poor prognostic factor of recurrence‐free survival in CRC.^17^ Inhibition of the extracellular terminus of PC1 reduces cell proliferation, suppresses EMT and promotes tumour necrosis in HT29 CRC xenografts. In renal cell carcinoma, PC1 favours angiogenesis and activates the phosphoinositide 3‐kinase (PI3K)/protein kinase B (AKT)/mTOR signalling cascade.^18^ Additionally, PC1 overexpression in hepatocellular cancer, lung cancer and CRC cell lines leads to the amplification of intercellular and ECM interactions. On the other hand, PC1 inhibition leads to increased cell proliferation and migration via the Wnt pathway.^41^ Overexpression of PC1 in the same cell lines is associated with a significant increase in apoptosis and cessation of the cell cycle in the G0 / G1 phase.^21^ In GOS‐3 GBM cells, PC1 was demonstrated to promote cell proliferation and migration, as well as upregulate mTOR signalling and downregulate JAK signalling.^19^

In this study, T98G GBM cells were subjected to HP in order to simulate the increased interstitial pressure that develops in GBM tumours. Under this condition, the extracellular mechanosensitive part of PC1 was blocked via the inhibitory antibody IgPC1 to evaluate whether and how PC1 functions in response to mechanical pressure in GBM cells. Our data show that the behaviour of GBM cells can be affected by HP and PC1. Specifically, HP seems to hinder GBM cell proliferation, migration and clonogenicity, while PC1 has the opposite effect on these oncogenic processes.

Additionally, using GBM spheroids, we displayed that both PC1 and HP may assist GBM cells to efficiently interact with each other, allowing them to stick together and form compact spheroids with a small diameter. Given the mechanotransductive properties of PC1, we sought to uncover signalling pathways and transcriptional regulators that function downstream of PC1 and are activated or inhibited in response to its functional blockade and HP. We found that PC1 and HP regulate the activation of signalling proteins mTOR, FAK and ERK, as well as the expression of transcription cofactors YAP and TAZ in GBM cells in a time‐dependent manner. Finally, we also provided evidence that PC1 and HP are implicated in the regulation of several cancer cell traits by affecting the expression of proteins related to apoptosis, anti‐apoptosis, angiogenesis, EMT and proliferation.

Overall, our in vitro findings highlight the role of PC1 in the mechanobiological mechanisms of GBM pathogenesis. Based on our work, GBM cells use PC1 to sense their mechanical microenvironment and respond to it by translating mechanical forces into biochemical signals that govern their oncogenic behaviour. Therefore, PC1 emerges as an additional mechanosensitive protein that participates in GBM development and progression, suggesting that it may represent a potential novel therapeutic target for this lethal brain cancer.^42^, ^43^ Further studies will provide a better understanding of the molecular underpinnings of the effects of mechano‐induced PC1 on GBM cells and will validate its role in the mechanobiology of GBM.

## CONFLICT OF INTEREST

The authors confirm that there are no conflicts of interest.

## AUTHOR CONTRIBUTION

**Ilianna Zoi:** Formal analysis (lead); Investigation (lead); Methodology (lead); Software (lead); Validation (equal); Writing – original draft (lead). **Antonios N. Gargalionis:** Conceptualization (equal); Data curation (equal); Formal analysis (lead); Funding acquisition (equal); Investigation (lead); Methodology (lead); Project administration (lead); Validation (equal); Visualization (equal). **Kostas A. Papavassiliou:** Data curation (lead); Formal analysis (lead); Validation (lead); Writing – original draft (lead). **Narjes Nasiri‐Ansari:** Formal analysis (equal); Investigation (equal); Software (equal); Validation (equal); Writing – original draft (equal). **Christina Piperi:** Conceptualization (equal); Resources (lead). **Efthimia K. Basdra:** Conceptualization (lead); Data curation (equal); Funding acquisition (equal); Resources (equal); Supervision (lead); Validation (lead); Visualization (equal); Writing – review & editing (equal). **Athanasios G. Papavassiliou:** Conceptualization (lead); Data curation (lead); Funding acquisition (lead); Resources (equal); Supervision (lead); Validation (lead); Visualization (lead); Writing – review & editing (lead).

## Data Availability

The data that support the findings of this study are available from the corresponding author upon reasonable request.
